# Nanohybrid of Thymol and 2D Simonkolleite Enhances Inhibition of Bacterial Growth, Biofilm Formation, and Free Radicals

**DOI:** 10.3390/molecules27196161

**Published:** 2022-09-20

**Authors:** Carlos Velázquez-Carriles, María Esther Macías-Rodríguez, Omar Ramírez-Alvarado, Rosa Isela Corona-González, Adriana Macías-Lamas, Ismael García-Vera, Adriana Cavazos-Garduño, Zuamí Villagrán, Jorge Manuel Silva-Jara

**Affiliations:** 1 Departamento de Farmacobiología, Centro Universitario de Ciencias Exactas e Ingenierías, Universidad de Guadalajara, Blvd. Marcelino García Barragán 1421, Guadalajara 44430, Mexico; 2 Departamento de Ingeniería Química, Centro Universitario de Ciencias Exactas e Ingenierías, Universidad de Guadalajara, Blvd. Marcelino García Barragán 1421, Guadalajara 44430, Mexico; 3 Departamento de Ciencias de la Salud, Centro Universitario de Los Altos, Universidad de Guadalajara, Av. Rafael Casillas Aceves 1200, Tepatitlán de Morelos 47600, Mexico

**Keywords:** terpenes, layered hydroxide salt, antimicrobial activity, biofilm-formation inhibition, antioxidant capacity

## Abstract

Due to the current concerns against opportunistic pathogens and the challenge of antimicrobial resistance worldwide, alternatives to control pathogen growth are required. In this sense, this work offers a new nanohybrid composed of zinc-layered hydroxide salt (Simonkolleite) and thymol for preventing bacterial growth. Materials were characterized with XRD diffraction, FTIR and UV–Vis spectra, SEM microscopy, and dynamic light scattering. It was confirmed that the Simonkolleite structure was obtained, and thymol was adsorbed on the hydroxide in a web-like manner, with a concentration of 0.863 mg thymol/mg of ZnLHS. Absorption kinetics was described with non-linear models, and a pseudo-second-order equation was the best fit. The antibacterial test was conducted against *Escherichia coli* O157:H7 and *Staphylococcus aureus* strains, producing inhibition halos of 21 and 24 mm, respectively, with a 10 mg/mL solution of thymol–ZnLHS. Moreover, biofilm formation of *Pseudomonas aeruginosa* inhibition was tested, with over 90% inhibition. Nanohybrids exhibited antioxidant activity with ABTS and DPPH evaluations, confirming the presence of the biomolecule in the inorganic matrix. These results can be used to develop a thymol protection vehicle for applications in food, pharmaceutics, odontology, or biomedical industries.

## 1. Introduction

Over the years, bacteria have developed resistance to several antibiotics, creating a severe problem in health sectors [1]. For this, the consumption of natural components that possesses bioactive compounds has been recommended, such as plant extracts or their essential oil [2]. Plant extracts have been studied due to their natural antioxidant, antimicrobial, anti-inflammatory, and antiseptic activities, as well as being precursors in the synthesis of pharmaceutical products [3,4].

The antibacterial and antioxidant activities of plant extracts can be attributed to phenolic compounds. Among these, terpenoids present in plants of the *Thymus* genus, including carvacrol and thymol, interact with the metabolic processes of bacteria [5]. Thymol is classified as generally recognized as safe (GRAS) and has been used in different food and pharmaceutical formulations [6] due to its antioxidant, antifungal, and antibacterial activities [7]. It has been proved that thymol in concentrations below 100 mg/mL exhibits antibacterial activity against Gram-negative and Gram-positive bacteria, such as *Escherichia coli*, *Staphylococcus aureus*, *Salmonella* spp., and *Pseudomonas aeruginosa*, including the inhibition of biofilm formation [1,8]. Even though this compound has special activities, it is also unstable to environmental factors, has poor solubility in water, and lacks a proper distribution on target sites [9].

Research in nanotechnology is currently been conducted in order to apply it in different areas, such as food science [10], electronics [11], and pharmaceutics [12]. In the latter, it has been proved that nanomaterials can retain pharmaceutical compounds to treat some types of cancer, demonstrating non-cytotoxic, antitumor activities [12].

Layered materials (LMs), or nanoclays, have increased their applications in biological fields due to their protection characteristics and controlled release of bioactive compounds. They can be classified as layered double hydroxides (LDH) and layered hydroxide salts (LHSs), with the difference being the presence of two metal or one metal in the structure, respectively [13]. LM can be synthesized through different pathways, such as precipitation, co-precipitation, or ion interchange; each method uses specific conditions, such as metallic salt concentration, temperature, alkaline solution applied, etc. [14] LM on its own may exhibit antibacterial activity with the formation of ROS that inhibit bacterial growth [15], but also, since LM forms hybrids, the intercalated molecule may generate higher inhibition to Gram-negative and Gram-positive bacteria, such as the antimicrobial peptide nisin [16], in which the effect was upgraded while the peptide was inside the LM. An example of LM is zinc hydroxide chloride, also known as Simonkolleite, a mineral found in galvanized steel that displays a layered structure that is brucite-like. In this structure, empty spaces are located below and above coordinated Zn, where counter-anions can be intercalated [17] such as cinnamate [18] or β-glucan [19]. Combining these LMs with bioactive compounds may exhibit equal or higher activities of the biomolecule, thus reducing toxicity due to smaller concentrations and production costs [19,20].

Even though thymol has been encapsulated in LDH [21], its intercalation in LHS has not been explored so far. This study focused on synthesizing a nanohybrid containing Simonkolleite (ZnLHS) and thymol and evaluating its antioxidant activity, antibacterial activity, and inhibition of exopolysaccharides formation of an opportunistic human pathogen such as *P. aeruginosa*, with which new materials could be applied in the health department.

## 2. Results and Discussion

### 2.1. Characterization

**Figure 1:**
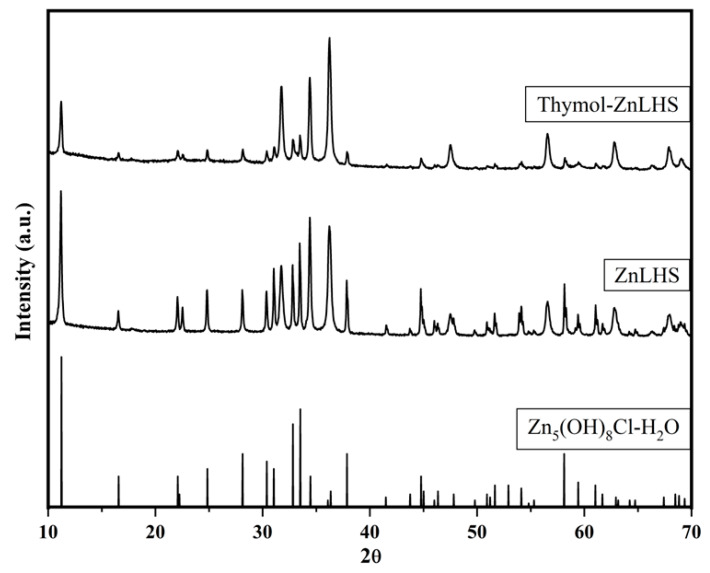
X-ray diffractograms of zinc layered hydroxide with and without thymol.

The X-ray diffractogram is depicted in Figure 1. It can be seen that Simonkolleite’s structure (Zn_5_(OH)_8_Cl_2_·2H_2_O) was obtained according to the ICDD card 07-0155 [16].

After intercalation, typical signals remained with reduced intensity, suggesting that thymol was not intercalated inside the interlaminar space but could be on the surface of the laminar compound [22]. To assume that the biomolecule was successfully intercalated inside the layered compound, the first peak should displace to smaller values of the 2θ angle, and interlaminar space could be calculated with Bragg’s Law [16]. Nevertheless, thymol crystallinity (diffractogram not shown) was diminished in thymol–ZnLHS, suggesting a strong interaction with the surroundings of Simonkolleite. A similar study was conducted for thymol nanoencapsulation, in which the XRD pattern of thymol disappeared in the nano-encapsulated thymol, suggesting the prevention of crystallinity, and this immobilization possibly promotes more reactive sites or confers beneficial applications for food products [23].

**Figure 2:**
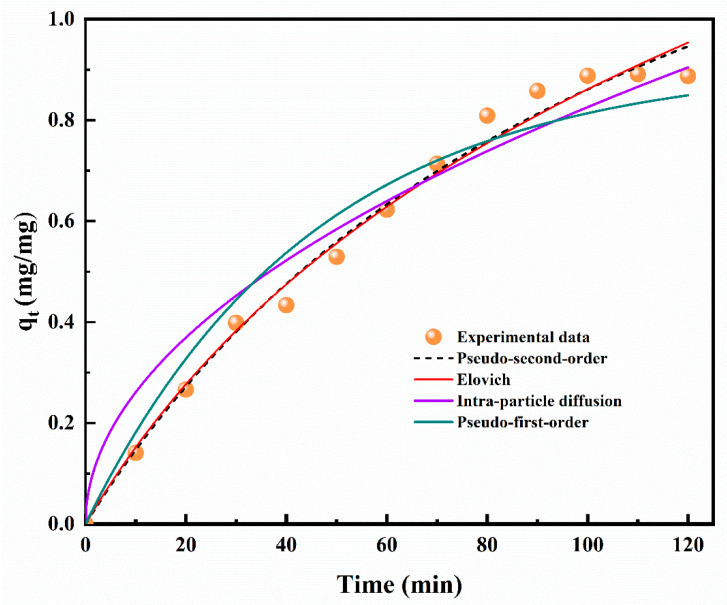
Thymol absorption in zinc layered hydroxide with non-linear fitted models.

The concentration of thymol adsorbed on the ZnLHS was determined by measuring the absorbance of the solution of intercalation. Previously, a standard curve was prepared to correlate the absorbance of thymol with the concentration. The equation from Bouaziz et al. [24] was applied, and the adsorption of thymol was estimated (Figure 2).

The adsorption phase was constant with a linear behavior (R^2^ = 0.989) until 1.5 h, and afterward, the absorbance remained constant, with a concentration of 0.863 mg thymol/mg ZnLHS. Thymol has been previously adsorbed in the LDH matrix of Mg-Al-CO_3_ with a maximum concentration of 8 mg/g in 4 h [21], while in hydroxyiron clays kaolinite and montmorillonite, the absorption reached values of 0.391 and 1.125 mg/mg, respectively, in 10 days [25]. Compared to the LDH matrix, in this study, the amount of thymol adsorbed was higher, probably due to the less complex structure that the LHS presents, and thus, the interaction could be favored [16]. Moreover, it has been reported that adsorption is time-dependent [21], but considering the concentration of thymol in clays and in the ZnLHS, values are not too different for their corresponding time of adsorption, and thus, the use of ZnLHS proved to be able to retain almost the same amount of biomolecule in less time.

Furthermore, in Figure 2, kinetic adsorption models are depicted, and in Table 1, the parameters *R*^2^, *RMSE*, *ARE*, χ^2^, *AIC*, and *BIC* are shown. The pseudo-second-order model proved to be the better fit with R^2^ = 0.989, and predictors exhibit the lowest values, followed by Elovich > pseudo-first-order > intra-particle diffusion model. The pseudo-second-order model describes that a chemisorption phenomenon was involved during the adsorption process, where valence forces acted through the exchange of electrons.

These processes have been previously described for polyphenol adsorption systems in the biomass of *Chlorella vulgaris* [26], in the adsorption of polyphenols in microporous starch [20], and in the adsorption process of polyphenols in roasted hazelnut skin [27], demonstrating a strong correlation.

**Figure 3:**
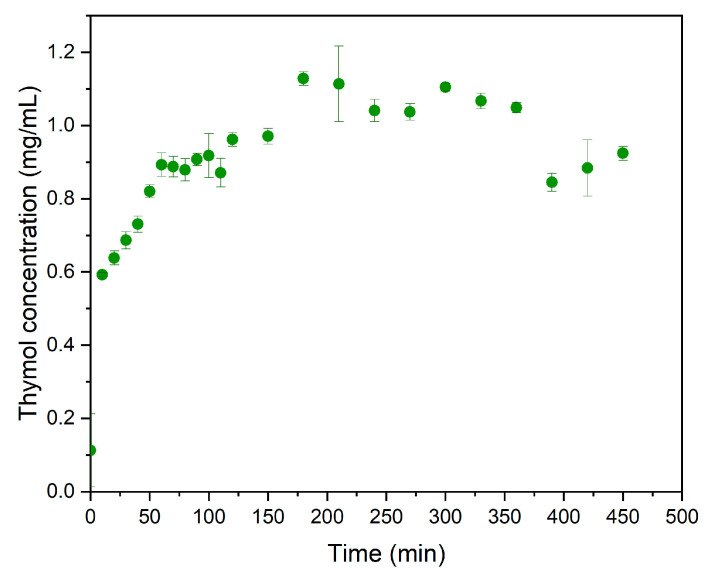
Thymol liberation from zinc hydroxide salt in PBS at 25 °C.

The liberation of thymol is depicted in Figure 3. It can be seen that, in the first hour, the thymol liberation rate was higher than it was during the rest of the test time, this can be attributed to rapidly achieving an equilibrium concentration of biomolecule adsorbed on the hydroxide surface. The thymol concentration kept increasing and decreasing with passing time, but always in a concentration range of 0.800–1.02 mg/mL, suggesting that zinc hydroxide kept the concentration in the state of equilibrium. This phenomenon may be helpful in controlling bacterial growth on surfaces since the bioactive compound would not be used only at the beginning, but it would constantly return to the inorganic matrix until the concentration of thymol falls off of equilibrium. In the study conducted by Guarda et al. [28], the liberation of thymol from microencapsulates was evaluated, and it can be seen that concentration of thymol was almost constant in a 28 days’ test, and the maximum of biomolecule concentration was liberated on the first day.

**Figure 4:**
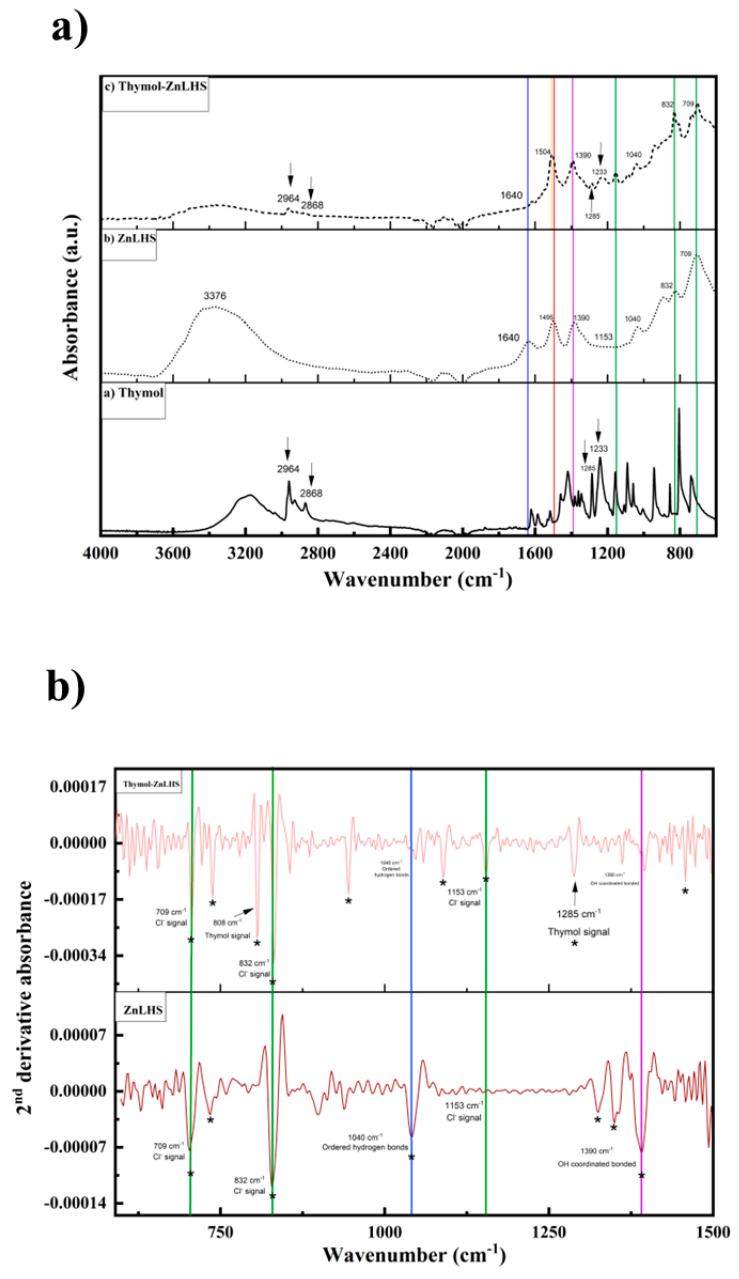
Fourier-transform infrared spectroscopy: (**a**) IR spectra of thymol and zinc layered hydroxide with and without thymol and (**b**) second derivative spectra of the analyzed samples in the 1500–600 cm^−1^ region; significant peaks were considered at *p* < 0.05 and represented by *.

FTIR spectra for ZnLHS, thymol, and thymol–ZnLHS are depicted in Figure 4. For thymol spectra (4a), characteristic signals can be found in the wavenumbers 2964, 2868, 1285, and 1233 cm^−1^, assigned to C=C stretching, -OH bending, and C-O stretching of phenolic compounds [29]. The ZnLHS FTIR spectra show O-H vibrations in the region of 3500–3000 and 1630–1250 cm^−1^, and the signal below 800 cm^−1^ corresponds to Zn-O bonds [16].

These signals appear in the thymol–ZnLHS spectrum (4c), with smaller intensities confirming the formation of the hybrid. Moreover, this phenomenon is found in other studies in which thymol was incorporated in a chitosan hydrogel [29,30]. Another important contribution can be identified in wavenumbers around 3376 cm^−1^, where the OH^−^ radicals’ signal that is present in the laminar compound (Figure 4b) is reduced in the hybrid, possibly due to the interaction with thymol [31].

**Figure 5:**
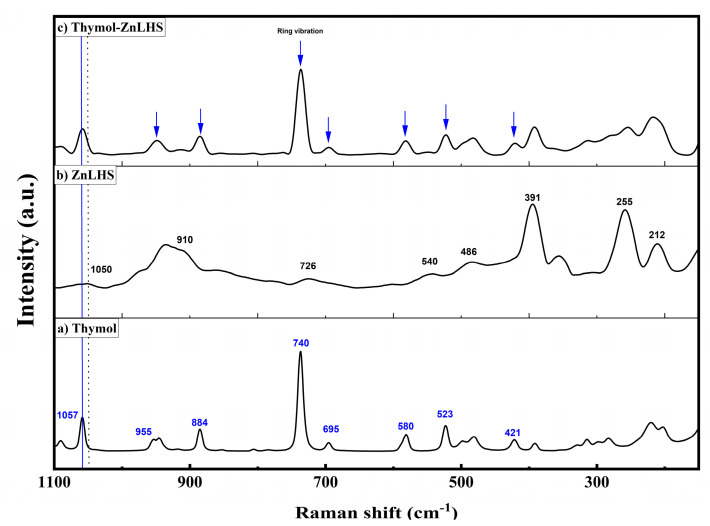
Raman spectroscopy of thymol, ZnHSL, and thymol–ZnHSL.

**Figure 6:**
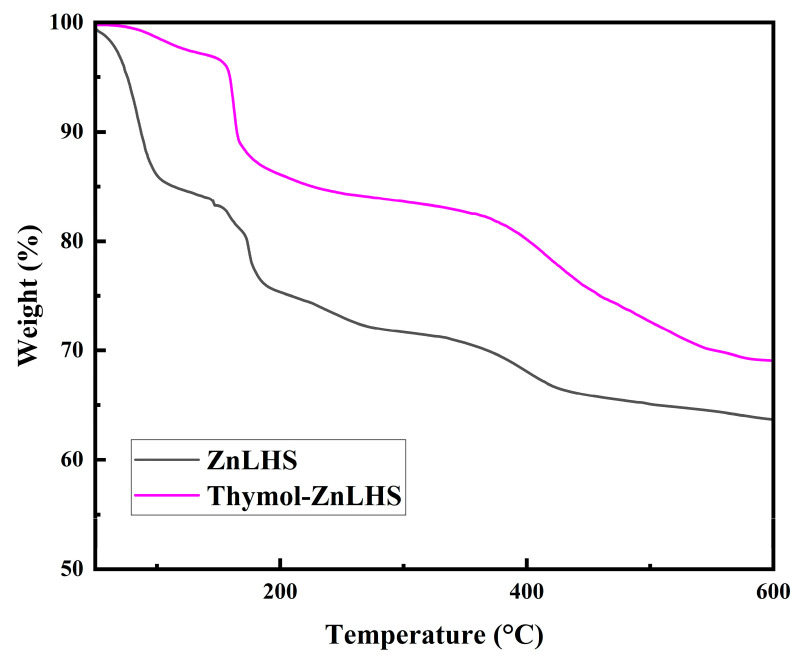
Thermograms of ZnHSL and thymol–ZnHSL.

Furthermore, small signals c.a. 2868, 1285, and 1233 cm^−1^ (wavenumbers with arrows), which are characteristic of the thymol spectrum, appear in the hybrid material, as reported in many studies [29,30]. According to Koosehgol et al. [32], even though the intensity of signals is not high, these small contributions can be assumed to be an indicator of the molecule’s presence, as found in the IR spectra of a chitosan–thymol hydrogel. Remarkably, a band located at 1640 cm^−1^ (blue line) in ZnLHS (spectrum b) is assigned to the δ(OH) vibrational mode of surface-bound water on the layered material. Spectrum c loses this vibrational mode, probably by the hydrogen-bonding or “ordered hydrogen bonds” interactions with thymol. Namely, some studies showed that the hydrogen bond extends to the sides of hydrophobic solutes and can be ordered as a network where, as far as is known, it can be cooperative and of electrostatic nature. Thus, interactions could be reached with neighborhood OH groups [33,34]. Moreover, the signal centered at 1495 cm^−1^ (spectrum b) is associated with the stretching of the C–C bonds from the aromatic rings and was slightly shifted on spectrum c at 1504 cm^−1^; probably, this fact explains the interaction of thymol with ZnLHS matrix (yellow vertical line on spectrum c). To elucidate more interactions, a second derivative of the region between 1500 and 600 cm^−1^ for ZnLHS and thymol–ZnLHS was obtained (Figure 4b); this spectrum clearly shows the explanations mentioned above, and in addition, an unresolved band centered at 1390 cm^−1^ in Figure 4a (violet vertical line) can be appreciated and resolved by second derivative (*p* < 0.05), advising the reduction of the OH coordinated bond [35]. Using the second derivative criterion proved to be significantly helpful in resolving weak and overlapping bands in the original spectra [36]. In the Raman spectra depicted in Figure 5a, thymol contributions are shown; specifically, a band toward 740 cm^−1^ was previously reported for the aromatic ring of thymol (SpectraBase^TM^ Wiley & Sons, 2022). On the other hand, in spectra 5b, signals for ZnLHS are shown, confirming the structure of Simonkolleite found in the database of the RRUFF project for minerals at 780 nm (ID R130117). Signals positioned in 212 and 391 cm^−1^ belong to Zn-O vibrations, while 255 cm^−1^ is assigned to the Zn-Cl vibration. Moreover, the signal around 1050 cm^−1^ is attributed to any intercalated anions [37]. In the thymol–ZnLHS spectra (Figure 5c), blue arrows mark positions related to thymol, thus confirming the presence of the terpene in the hybrid structure. Even signals around 1058 cm^−1^ in spectrum a (blue line) and 1050 cm^−1^ in spectrum b (dotted line) are slightly displaced, suggesting a joint vibration in thymol–ZnLHS. Interestingly, signal toward 255 cm^−1^ for thymol–ZnLHS diminishes its intensity compared to ZnLHS, adverting the vibration of the chloride anion present in the interlamellar structure. Furthermore, the 1053 cm^−1^ region belonging to the thymol aromatic ring in spectrum c shows a slight displacement, a behavior that has been previously reported due to hydrophobic interactions [37]. These facts sustain findings in the vibrations of IR spectra. Thermograms of ZnHSL and thymol–ZnHSL are depicted in Figure 6. For layered hydroxide, similar behaviors have been previously reported [16,19]. On the other hand, thymol–ZnHSL exhibits a first event around 150 °C, where a 10% mass is lost, and that could be related to the thymol surrounding the hydroxide, since this terpene has been reported to reduce its mass drastically between 150 and 200 °C [38]. The next event comes near 400 °C, where Cl- and OH- interlaminar anions degrade, and finally, there is a total oxidation to ZnO in temperatures above 500 °C [16].

Therefore, the XRD, FTIR, Raman, and TGA techniques suggest that the thymol molecule was adsorbed. However, interestingly, signals located at 1153, 832, and 709 cm^−1^ (green vertical lines in Figure 4a,b) warn about the presence of a Cl^−^ ions vibration, which is a counterion present in the typical Simonkolleite interlamellar space. The signal is diminished at 709 cm^−1^ for the nanohybrid spectrum and could suggest possible partial intercalation [39]. In this type of reaction, chlorine reacts with phenol or compounds containing phenolic groups such as thymol and can then support the displacement of the C-C aromatic ring band [40].

**Figure 7:**
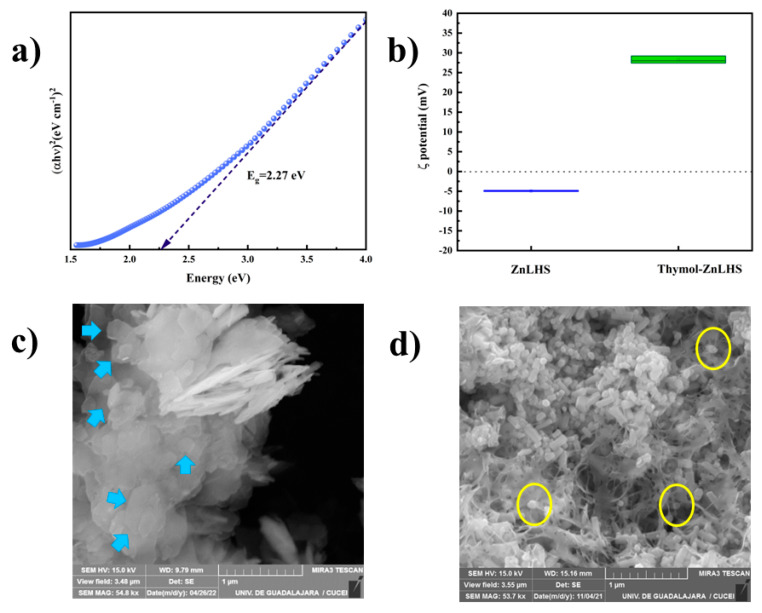
Optical energy plot and zeta potential. (**a**) Bandgap of Simonkolleite (ZnLHS) and (**b**) ζ-potential of Simonkolleite (ZnLHS) and thymol–ZnLHS, respectively. Scanning electronic microscopy: (**c**) ZnLHS and (**d**) thymol–ZnLHS.

The insertion or removal of water molecules causes changes in the electronic structure (something not so familiar in 2D-type structures), generating possibilities to function in different areas of knowledge by its photoelectronic properties [41]. A recent publication by Baig et al. [15] found a reduced bandgap (1.8 eV) attributed to the antibacterial action of pristine LHS by ROS species’ generation. To observe this phenomenon, we calculated a bandgap of Simonkolleite studied here by Tauc’s relation, using a UV–Vis spectrophotometer (Optizen Pop, K LAB). The bandgap analysis (Figure 7a) revealed a small value (2.27 eV), but the value was slightly higher compared with that of the authors. Reduced bandgap values are related to the presence of chloride ions in the zinc matrix, producing a heterojunction between the valence band and the conduction band, which gives it potential photocatalytic properties in visible wavelength ranges [15].

The zeta potential (ζ) influences the stability of the particles through electrostatic repulsions. For stable dispersions, values must be greater than ±30 mV, considering that values above this value are less sensitive to agglomerations or destabilization caused by van der Waals forces or Brownian motion [42]. The ζ-potential analysis showed that laminar compounds (ZnLHS and thymol–ZnLHS) exhibited a negative potential of −4.93 ± 0.14 and a positive potential of +29.20 ± 0.90, respectively (Figure 7b). It is known that negative zeta potential values are associated with the accumulation of positive charges surrounding the nanomaterial, giving it a negative nature [43]. In other studies, this increase in ζ-potential has been demonstrated to be an effect of thymol in silica carrier agents [44]. A possible explanation could be associated with the hydrophobicity of both materials maintaining a repulsion electrostatic in the media. Moreover, this phenomenon was correlated in a study conducted by Mattos et al. [45] in which uncharged thymol added to the silica matrix showed positive and negative electrostatic potential, shifting values to zero in a pH-dependent manner. The mean particle size was reduced after intercalation from 589.80 ± 18 nm to 141.05 ± 13.85 nm, and the polydispersity index was also diminished from values between 0.58 ± 0.09 and 0.33 ± 0.05. This decrease improves the size and morphology distribution of the hybrids. Several methods to synthesize nanoclays lack homogeneity in their morphology, which can be a desirable property for applications in the food and pharmaceutical industries [46,47].

Micrographs depicted in Figure 7c exhibit the typical hexagonal morphology (blue arrows) of layered compounds (at least one dimension in the nano-range order) [48]. In the hybrid (Figure 7d), it can be seen that the structure remained, but the size decreased, and the organic part surrounding the particle (yellow circles), in some cases, formed networks of ZnLHS and biomolecule. Similar results were obtained by the interaction of glucans with a zinc–hydroxy chloride [16]. Furthermore, the decrease in the particle size was studied by Gutiérrez-Gutiérrez et al. [22] when curcumin was loaded into layered compounds to avoid its agglomeration, and the ζ-potential values supported this fact.

### 2.2. Antioxidant Activity

**Figure 8:**
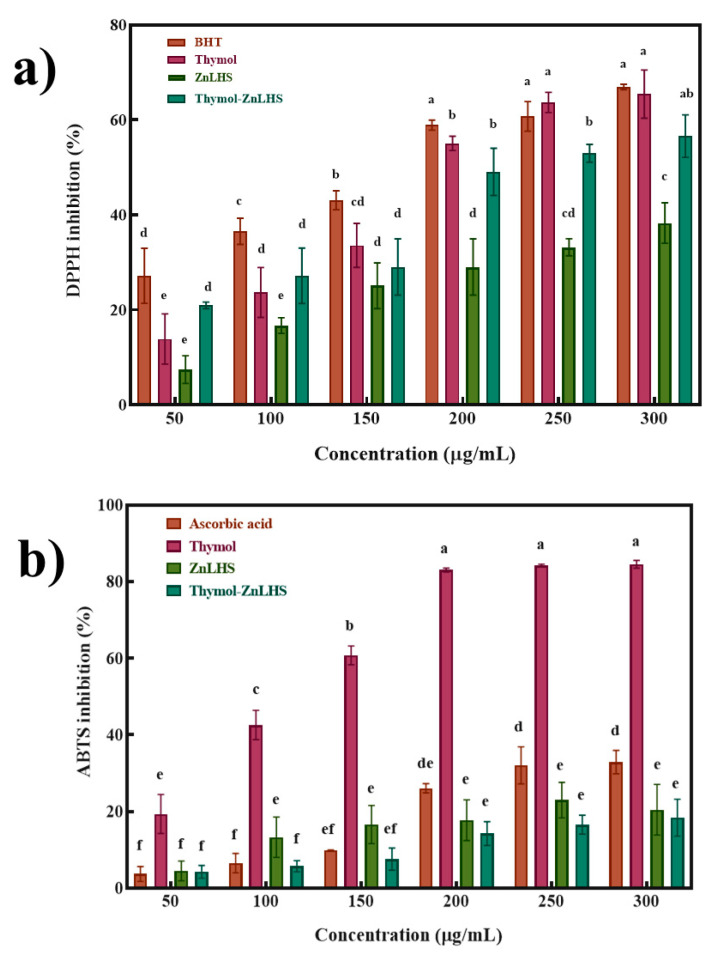
Antioxidant capacity of thymol, ZnLHS, and thymol–ZnLHS: (**a**) DPPH inhibition (%) and (**b**) ABTS inhibition (%). For DPPH and ABTS assays, different letters are significantly different at a *p* < 0.05.

The DPPH and ABTS tests were carried out to determine the antioxidant activity of the thymol–ZnLHS hybrid (Figure 8a,b). The results demonstrated that thymol–ZnLHS exerts slightly more ABTS activity than ZnLHS alone, thus confirming the biomolecule in the hybrid. For the DPPH assay, thymol–ZnLHS exhibited higher activity than thymol and ZnLHS at low concentrations, but the effect was inverted at high concentrations. A study performed by Rúa et al. [2] found that high concentrations of other compounds, such as carvacrol, in the extract solutions inhibit the antioxidant activity of thymol. Since the ZnLHS exhibited antioxidant activity by itself, this capacity to generate ROS species related to the reduced bandgap [15] could have reduced the activity of thymol compared to the control. According to Deng et al. [49], the solubilization of thymol could increase with different solvents, but the antioxidant effect could be lost; apart from that, if incorporated into a food matrix, the flavor could be altered.

### 2.3. Antibacterial Activity

Inhibition halos were measured after incubation and are shown in Table 2.

The diameters were higher for *S. aureus* than for *E. coli* O157:H7. Thymol is an antibacterial compound that exhibits antibacterial activity against both Gram-negative and Gram-positive bacteria. Possibly, the higher surface charge (zeta-potential) will increase the interaction between cells/nanoparticles, leading to a better transfer of thymol; it allowed the molecule to exhibit higher inhibition halos for the hybrid than the biomolecule alone [50,51]. Xu et al. [52] determined the inhibitory concentration of thymol, with values around 200 mg/mL for *E. coli*. Compared to this study, the inhibitory concentration was observed in lower concentrations. Palygorskite functionalized with thymol achieved better antimicrobial properties against *S. aureus* than thymol alone since the improved hydrophilic character of the composite promotes the transport of monoterpene in clays [53]. Likewise, in another study, the antimicrobial action was verified through another system composed of clinoptilolite–zeolite clays loaded with thymol or carvacrol, which showed more extraordinary antimicrobial properties against *E. coli* and *S. aureus*. Inhibition was not only attributed to the release of monoterpenes but also to the enhanced activity, as new properties such as hydrophilia of the hybrids were introduced [54]. Moreover, the stability of nanomaterials as colloids is related to high values of ζ-potential (negatively or positively charged), promoting stability to the dispersion and, thus, improving bactericidal efficacy [55]. In addition, the reduced bandgap value may confer the capacity in these materials to kill bacteria through its ROS release in a synergistic manner with ζ-potential; all of these findings align with the work published by Baig et al. [15].

### 2.4. Inhibition of Biofilm Formation

**Figure 9:**
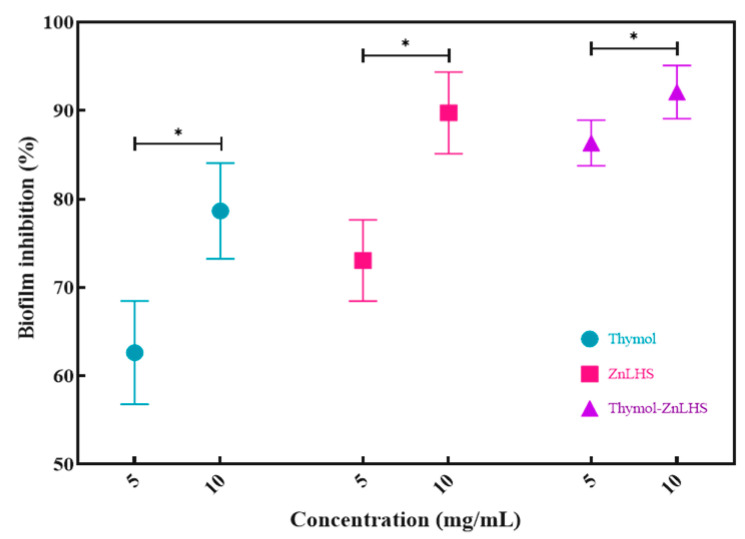
Biofilm inhibition of *Pseudomonas aeruginosa* (%). Significant differences were based on LSD Fisher post hoc paired comparisons (* *p* < 0.05).

According to several authors, pathogenic biofilm is relevant to health since it can lead to severe illness or even death [51]. The percentage of inhibition of biofilm formation is found in Figure 9.

Inhibition for ZnLHS at 5 and 10 mg/mL was of 73 and 89%, respectively, while for thymol, the results with the same concentrations were of 62 and 78%. Interestingly, the thymol–ZnHSL hybrid increased the inhibition of biofilm formation in 86 and 92% for each concentration tested, demonstrating a synergistic behavior of layered hydroxide and terpene molecules. The percentage of inhibition was statistically different for each material and concentration (*p* < 0.05).

Common disinfectants oxidize the cell membrane before biofilm forms [56]; this suggest that the hydroxide salt, due to its anionic nature, can inhibit this polysaccharide synthesis. Moreover, it has been proved that thymol suppresses biofilm-associated genes, and for that, the combination of both compounds may increase the inhibition rate of biofilm formation [57]. Hydroxide salt, anionic nature oxidizes, and thymol suppress biofilm-associated genes.

The increase in the percentage of inhibition presented by the thymol–ZnLHS compared to thymol alone may be because the latter has a relatively hydrophilic character that, when stabilized in colloidal dispersion, can favor its diffusion through the polysaccharide matrix with polar character. On the contrary, the hydrophobic character of ZnLHS could interact specifically with the bacterial membrane (behavior also observed by thymol). Therefore, the nanohybrid increases inhibition due to a synergistic effect [58]. This synergic effect has been reported previously with other compounds, such as nalidixic acid/zinc hydroxide nitrate [59].

## 3. Materials and Methods

### 3.1. Synthesis of ZnLHS and Thymol–ZnLHS

The synthesis of materials and hybrids was conducted by following the methodology described by Velazquez-Carriles et al. [16]. Briefly, 200 mL of a solution containing 0.04 g/mL ZnCl_2_ was prepared and allowed to stabilize at room temperature, with constant stirring, for 20 min. Then the pH (Hanna, HI98115) was gradually elevated by adding NaOH 0.1 M dropwise until it reached a final value of 8 and a white precipitate formed. The solution was covered and allowed to stabilize for 24 h at room temperature, with constant stirring. Solids were recovered by centrifugation (10,000 rpm for 10 min at 25 °C) (LaboGene, LZ-1580R) with three consecutive washes with distilled water. The recovered powder (ZnLHS) was then dried in an oven (L-C Oven, Mechanically Convected) at 60 °C for 24 h and reserved until use.

For the hybrid synthesis, a solution of 5 mg/mL of thymol was prepared, and 500 mg of ZnLHS was added, with constant stirring at room temperature. Aliquots of supernatant were evaluated on a UV–Vis (Nanodrop 2000, ThermoScientific), at a wavelength of 274 nm, every 10 min, until constant absorbance was achieved (ca. two hours). The amount of thymol interacting with the ZnLHS was estimated with the formula proposed by Bouazis et al. [24] For recovery of the hybrid thymol–ZnLHS, the conditions mentioned above for centrifugation and drying were used.

The results of adsorption kinetics were fitted with four models: pseudo-first-order, pseudo-second-order, Elovich, and intra-particle diffusion. The best model was selected with respect to the highest correlation coefficient, R^2^, and also with the lowest in the following five statistical parameters: root mean squared error (*RMSE*), average relative error (*ARE*), chi-square (x^2^), Akaike information criterion (*AIC*), and Bayesian information criterion (*BIC*). Each parameter was calculated with the following equations:

(1) $$RMSE=\sqrt{\frac{\sum_{i=1}^{N}{\left(q_{t,predicted}-q_{t,exp}\right)}^{2}}{N}}$$

(2) $$ARE=\frac{100}{N}\sum_{i=1}^{N}[\frac{q_{t,exp}-q_{t,predicted}}{q_{t,exp}}]$$

(3) $$\chi^{2}=\sum_{i=1}^{N}\frac{{\left(q_{t,exp}-q_{t,predicted}\right)}^{2}}{q_{t,predicted}}$$

(4) $$AIC=\frac{-2}{N}*LL+2*\frac{k}{N}$$

(5) BIC=−2*LL+log(N)*k

where *N* is the number of experimental data, *q_t,predicted_* is the calculated value in mg/mg with each kinetic model, and *q_t,exp_* is the experimental value (mg/mg). *LL* is the logarithmic likelihood, and *k* is the number of parameters involved in the model.

For thymol liberation, 60 mg of thymol–ZnHSL was suspended in 50 mL of PBS at 25 °C (approximately 1.02 mg thymol/mL), with constant stirring, and aliquots of supernatant were taken to measure the thymol concentration in a UV–Vis spectrophotometer at 274 nm until constant value was achieved.

### 3.2. Characterization

X-ray diffractograms (XRDs) were collected in an Empyrean X-ray Diffractor (Panalytical, Malvern, UK), using CuKα radiation at an angle 2θ between 5 and 70°, with a 0.02 step and 30 s of collection time. Fourier-transform infrared spectra (FTIR) were recorded in a spectrophotometer (Cary 630, Agilent Technologies, Santa Clara, CA, USA) from 4000 to 500 cm^−1^ in absorbance mode, with 32 scans and 4 cm^−1^ of resolution. To determine the morphology, scanning electron microscopy (SEM) was applied in an FE-SEM (TESCAN, model MIRA 3 LMU, Brno, Czech Republic), with a voltage of 15 kV. Particle size, polydispersity index, and ζ-potential were determined by dynamic light scattering (DLS) in a Zetasizer Nano ZS90 (Malvern Instruments, Malvern, UK) at 25 °C and a pH of 7, adding 1 mL of a diluted solution of 1 mg/mL (10-fold) ZnLHS and thymol–ZnLHS. High-resolution Raman spectroscopy was conducted in a SmartRaman (DXR2, Thermo Fisher Scientific, Waltham, MA, USA), with 780 nm laser excitation, 50 mW, and a slit of 50 µm; the acquisition time was 150 s at 3 cm^−1^ of resolution. The spectra were recorded between 1100 and 150 cm^−1^. Thermogravimetric analyses were carried out on a Discovery thermobalance (TGA5000, TA Instruments, New Castle, DE, USA). TG curves were registered by heating sample masses from 50 to 600 °C, using a ramp of 10 °C min^−1^, under a nitrogen atmosphere.

### 3.3. Antioxidant Activity

The antioxidant activity of ZnLHS and thymol–ZnLHS was determined with 2,2′-azino-di-(3-ethylbenzthiazoline sulfonic acid (ABTS) and 2,2-diphenyl-1-picrilhidrazil (DPPH) tests, comparing with common antioxidant molecules, as well as thymol standard (Sigma Aldrich, St. Louis, MO, USA). For ABTS, Li et al.’s [60] methodology was followed, preparing solutions of the samples at different concentrations (50–300 μg/mL), using ascorbic acid as a positive control and methanol as a negative control. Inhibition of radical DPPH was conducted as described by Brand-Williams et al. [61], with sample solutions at the same concentrations mentioned before; the positive control was BHT, and the negative control was methanol. The results are expressed as a percentage of inhibition for both tests. Plates were read at 754 nm for ABTS and 520 nm for DPPH in a 96-well microplate reader (BIO-RAD, iMark, Hercules, CA, USA). In both techniques, the following formula was employed:

(6) $$Inhibition\;\left(\%\right)=\left(\frac{Abs_{control}-Abs_{sample}}{Abs_{control}}\right)*100$$

where $Abs_{control}$ is the absorbance of control that contains all the reagents, except the samples.

### 3.4. Antimicrobial Evaluation

The inhibition halo test was conducted to assess the antibacterial activity of hybrids. Briefly, Müller–Hinton agar (MHA) plates were prepared, and 100 μL of cell suspension of *E. coli* O157:H7 and *S. aureus* ATCC 25,923 at 1 × 10^8^ cell/mL was spread on the surface. In each plate, holes were bored, and 100 μL of thymol, ZnLHS, and thymol–ZnLHS in a range of 1 to 10 mg/mL in PBS was added. Plates were incubated for 24 h at 36 ± 1 °C, and the inhibition zone was measured in mm.

### 3.5. Antibiofilm Activity

Some bacteria produce a biofilm to protect themselves against toxic compounds. A test following the methodology of O’Toole (2011) was applied to determine the inhibition of biofilm formation. Briefly, a culture of *Pseudomonas aeruginosa* ATCC 27,853 was grown overnight at 36 ± 1 °C in Luria Bertani broth and then diluted at 1:100 in the same fresh medium containing arginine as a carbon source and magnesium sulfate. In 96-well plates, 100 μL of *P. aeruginosa* culture was added, followed by 20 μL of thymol, ZnLHS, or thymol–ZnLHS solutions at 5 and 10 mg/mL (previously established with antimicrobial evaluation); as the negative control, PBS was used. The plate was incubated for 24 h at 36 ± 1 °C; then the culture was discarded, and the plate was washed in a deionized water bath twice and allowed to dry at room temperature. Subsequently, 125 μL of a solution of crystal violet (0.1% in water) was added to all wells and incubated at room temperature for 15 min. Plates were washed and dried as mentioned before. After the plate was dried, 125 μL of acetic acid (30% in water) was added, and the plate was incubated for 15 min at room temperature. Finally, volumes were transferred to a new plate, and the absorbance was measured at 550 nm in a 96-well plate reader (BioRad, iMark, Hercules, CA, USA), using acetic acid as blank. The inhibition of biofilm formation was calculated with the following equation:

(7) $$\%biofilm\;inhibition=\left(1-\frac{(Abs_{sample}-Abs_{blank)}}{\left(Abs_{control}-Abs_{blank}\right)}\right)*100$$

where (*Abs_control_*) is the absorbance of control that contains all the reagents, except the materials or thymol.

### 3.6. Statistical Analysis

All the experiments were performed in triplicate (±SD). Significant differences were considered at *p* < 0.05, using ANOVA, followed by Fisher’s LSD test (Statgraphics Centurion XIX, Princeton, NJ, USA).

The second derivative for the region between 600 and 1500 cm^−1^ of the FTIR spectra was analyzed, using 9 points, by the Savitzky–Golay algorithm. Then, to compare the absorbances intensities between ZnLHS and thymol–ZnLHS, the normality examination was applied (Anderson–Darling, D’Agostino–Pearson omnibus, and Shapiro–Wilk tests) in consequence (normality rejection), and a Mann–Whitney U test was established to determine wavenumbers significantly (*p* < 0.05). Origin 2022 (OriginLab Inc., Northampton, MA, USA) version was employed, and GraphPad Prism v8.0.1 (Dotmatics, San Diego, CA, USA) was used.

## 4. Conclusions

A hybrid of zinc layered hydroxide salt and thymol with biological activity was successfully synthesized. Characterization with X-ray diffraction confirmed a Simonkolleite structure for the layered compound; IR and Raman spectra and SEM micrographs showed that the organic compound surrounded the inorganic materials, mainly. The thermogravimetric analysis determined that the material could undergo temperatures around 200 °C before losing thymol due to degradation. Adsorption kinetics was described with non-linear models with R^2^ of 0.989 and a concentration determined by UV–Vis absorbance of 0.863 mg thymol/mg ZnLHS, and the liberation in PBS demonstrated that thymol can be released almost totally in about 4 h. The biological activity was tested with antioxidant and antimicrobial tests. ABTS and DPPH demonstrated that the hybrid synergistically exhibits antioxidant activity. For antimicrobial activity, Gram-positive bacteria such as *Staphylococcus aureus* were more sensitive to exposition to the hybrid of thymol and zinc hydroxide layered salt in low concentrations. Biofilm formation of *Pseudomonas aeruginosa* was almost completely inhibited with the hybrid compared to the materials alone. The findings of this work demonstrate a promising role for this nanohybrid as a decolonizing agent and preventive agent, making it attractive in various areas, such as food science, pharmaceuticals, dentistry, and clinics, helping to keep society healthy. Finally, we can infer that this nanohybrid could belong to a new generation of compounds with advanced biological properties.

## Figures and Tables

**Table 1:** Non-linear kinetic parameters with the correction coefficients for thymol adsorption on ZnLHS.

Kinetic Model	Statistical Validation					
	*R* ^2^	*RMSE*	*ARE*%	χ^2^	*AIC*	*BIC*
Lagergren’s pseudo-first order	0.954	0.060	15.727	0.092	−64.464	−65.436
Ho and McKay’s pseudo-second order	0.989	0.031	4.654	0.017	−81.752	−82.724
Elovich	0.986	0.033	4.431	0.019	−80.157	−81.129
Intra-particle diffusion	0.950	0.065	15.636	0.131	−62.407	−63.379

*R*^2^, correlation coefficient; *RMSE*, root mean squared error; *ARE*%, average relative error; χ^2^, Chi-square; *AIC*, Akaike information criterion; *BIC*, Bayesian information criterion.

**Table 2:** Inhibition halo of *E. coli* O157:H7 and *S. aureus* ATCC 25,923 (SD ± 3).

Concentration (mg/mL)	*Escherichia coli* (mm)			*Staphylococcus aureus* (mm)		
	Thymol	ZnHSL	Thymol–ZnLHS	Thymol	ZnHSL	Thymol–ZnLHS
**1**	10.3 ± 0.1 ^f^	10.3 ± 0.2 ^f^	11.0 ± 0.5 ^e^	10.3 ± 0.6 ^F^	10.5 ± 0.2 ^F^	16.2 ± 2.3 ^D^
**3**	10.6 ± 0.5 ^f^	10.6 ± 0.5 ^f^	10.9 ± 0.2 ^e^	11.8 ± 0.4 ^F^	10.5 ± 0.3 ^F^	19.5 ± 0.5 ^C^
**5**	21.2 ± 0.1 ^c^	10.4 ± 0.5 ^f^	11.7 ± 0.6 ^e^	13.0 ± 1.0 ^E^	10.4 ± 0.4 ^F^	20.7 ± 0.6 ^B^
**7**	22.4 ± 0.4 ^b^	10.2 ± 0.3 ^f^	15.5 ± 0.5 ^d^	19.3 ± 1.5 ^C^	10.2 ± 0.3 ^F^	22.7 ± 0.8 ^A^
**10**	23.9 ± 0.3 ^a^	10.7 ± 0.3 ^e^	21.1 ± 0.1 ^c^	21.2 ± 0.7 ^B^	10.7 ± 0.6 ^E^	24.0 ± 1.0 ^A^

Different superscripts indicate significant (*p* < 0.05) difference among groups. Lower and Upper case are to differentiate between bacterial strains.

## Data Availability

The data that support the findings of this study are available from the corresponding author upon reasonable request.

## Sample Availability

Samples of the compounds are not available from the authors.
